# Linking Microscopic Spatial Patterns of Tissue Destruction in Emphysema to Macroscopic Decline in Stiffness Using a 3D Computational Model

**DOI:** 10.1371/journal.pcbi.1001125

**Published:** 2011-04-21

**Authors:** Harikrishnan Parameswaran, Arnab Majumdar, Béla Suki

**Affiliations:** Department of Biomedical Engineering, Boston University, Boston, Massachusetts, United States of America; University of California San Diego, United States of America

## Abstract

Pulmonary emphysema is a connective tissue disease characterized by the progressive destruction of alveolar walls leading to airspace enlargement and decreased elastic recoil of the lung. However, the relationship between microscopic tissue structure and decline in stiffness of the lung is not well understood. In this study, we developed a 3D computational model of lung tissue in which a pre-strained cuboidal block of tissue was represented by a tessellation of space filling polyhedra, with each polyhedral unit-cell representing an alveolus. Destruction of alveolar walls was mimicked by eliminating faces that separate two polyhedral either randomly or in a spatially correlated manner, in which the highest force bearing walls were removed at each step. Simulations were carried out to establish a link between the geometries that emerged and the rate of decline in bulk modulus of the tissue block. The spatially correlated process set up by the force-based destruction lead to a significantly faster rate of decline in bulk modulus accompanied by highly heterogeneous structures than the random destruction pattern. Using the Karhunen-Loève transformation, an estimator of the change in bulk modulus from the first four moments of airspace cell volumes was setup. Simulations were then obtained for tissue destruction with different idealized alveolar geometry, levels of pre-strain, linear and nonlinear elasticity assumptions for alveolar walls and also mixed destruction patterns where both random and force-based destruction occurs simultaneously. In all these cases, the change in bulk modulus from cell volumes was accurately estimated. We conclude that microscopic structural changes in emphysema and the associated decline in tissue stiffness are linked by the spatial pattern of the destruction process.

## Author Summary

Current standards for characterizing microscopic structural changes in emphysema are based on estimating the amount of tissue loss using stereological techniques. However, several previous studies reported that, in emphysema, there is a lack of correlation between stereological indices of tissue structure and increases in lung compliance, which is the inverse of tissue stiffness. In this study, we developed a novel three-dimensional computational model to show that the amount of tissue loss is not the sole determinant of increased lung compliance in emphysema. A key component that needs to be considered is the pattern of tissue destruction, which we demonstrate has a significant effect on the rate of decline in stiffness. Our findings also indicate that the heterogeneity observed at the microscopic scale in emphysema is a signature of the spatial history of the destruction process. These results highlight the importance of characterizing the heterogeneity of lung tissue structure in order to be able to relate microscopic structural changes to macroscopic functional measures such as lung compliance.

## Introduction

Emphysema is a chronic obstructive pulmonary disease (COPD) that commonly occurs in conjunction with chronic bronchitis. While tobacco smoke is believed to be the primary risk factor for emphysema [1], other factors such as environmental pollutants [2], senescence [3], [4], nutrition [5]– and genetic predispositions [8] can also cause emphysema. Each of these risk factors triggers a series of interconnected biochemical processes that lead to cell death and the degradation of protein fibers that reinforce alveolar walls. Consequently, alveolar walls rupture and abnormally enlarged airspaces appear. Over time, the destruction of alveolar walls become progressive and patients experience increased difficulty in breathing [1]. In clinical settings, doctors rely on spirometric indices such as the amount of air that can be forcefully exhaled in 1 second (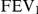) to detect and characterize the progession of this disease [9]. However, significant destruction of tissue can occur at the microscopic scale before emphysema can be detected using 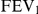 [10]. In order to develop better diagnostic methods and to understand how emphysema progresses, it is essential to first understand the link between microscopic changes in structure and global measures of function.

Emphysematous patients differ widely in the rate of decline in lung function as well as their morphometric characteristics. Despite this apparent heterogeneity, there is data suggesting that different morphological changes affect differentially lung function. For instance, in smokers, the decline in lung function is faster for patients with lesions concentrated in the upper zones of the lung as compared to patients with a more uniform destruction pattern [11]. In patients with 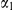-antitrypsin deficiency, a rare genetic form of emphysema, 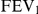 was found to correlate better with the extent of destruction evaluated from CT when the destruction is in the basal part of the lung [12], [13]. Further, these macroscale patterns have also been linked to different microscale structures [14], which in turn, have also been found to influence functional parameters such as lung compliance [15]. These findings suggest that there is a possible link between emphysema pathology, patterns of tissue destruction and decline in lung function. However, such a relationship between microscopic patterns of destruction and loss of function has not been identified.

**Figure 1:**
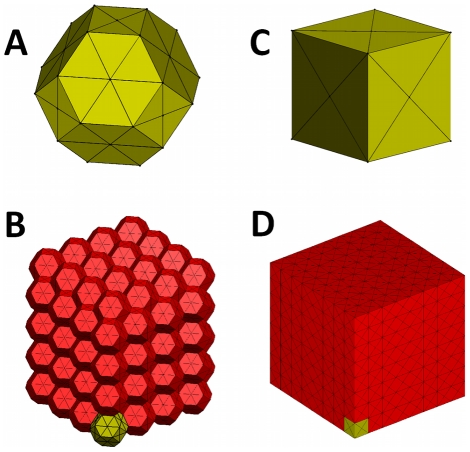
Initial geometry of a cuboidal block of tissue built by tiling space filling polyhedral cells representing idealized alveoli. Two types of space filling solids were used to approximate alveoli: tetrakaidecahedra (14-hedra) and cubes (A & C) show the individual unit cells. Elastic properties of the alveolar wall were modeled by face springs connecting the center of each face to its vertices. Additionally, springs were also placed along the edges of the unit cell. Note that shading is applied for better visualization. (B & D) show the three dimensional network obtained by tiling the unit cells.

Previous studies have shown that lung compliance can vary significantly even when structural measurements made from two dimensional (2D) sections of the lung did not show any significant change [15], [16]. This apparent lack of structure-function relationship maybe due to the fact that 2D sections do not accurately represent the true three-dimensional (3D) geometry of lung tissue and are significantly more error prone because of the noise introduced by sectioning [17]. Indeed, recent direct measurement of 3D alveolar structure has shown that the actual size of an alveolus measured in 3D is 1.5 times its current 2D estimate [18]. Therefore, the problem of relating structural and functional changes in emphysema is best analyzed in 3D. In this study, we developed a 3D model of lung tissue which incorporates the interdependent nature of the lung parenchyma– where upon destruction of an alveolar wall, the microscopic structure of tissue rearranges to balance forces [19]. Specifically, we modeled a block of lung tissue as a tessellation of space filling polyhedra, with each of the unit cells representing an idealized alveolus (Fig. 1). The model can be pre-strained and various destruction patterns can be mimicked by eliminating walls in a spatially random or in a correlated manner in which elimination is based on force carried by a face.

## Results

We considered a 3D elastic structure formed by tessellating space filling polyhedral unit cells which mimic alveolar airspace units in the lung tissue. The entire structure is pre-strained and its boundaries fixed. We used two kinds of space filling polyhedra: tetrakaidecahedra (hereafter referred to as “14-hedra”) and cubes (the simplest platonic space filling solid) to mimic the initial geometry of the alveolar spaces (Fig. 1). The elastic properties of the alveolar walls are represented by springs connecting the center of each face to its vertices. The edges of the unit cells also contain springs which represent the junction of septal walls. To simulate tissue destruction in emphysema, we removed faces that separate adjacent polyhedra. This involves breaking the face springs that span the corresponding face and calculating the new equilibrium configuration by minimizing the total energy of the system. When all the walls adjoining an edge are eliminated, the corresponding edge spring is also considered broken. We assume that the initial seed of the destruction process is spatially random, however, any further destruction follows one of the following patterns.

### 

#### Spatially random destruction

In this method, the faces selected for removal at every step were randomly chosen from the network. It is important to note that in this case, the continued destruction of walls is independent of the history of breaking. This method mimics the biochemical process where inhaled particles such as those found in cigarette smoke randomly trigger interstitial cells to release enzyme that cleave the ECM fibers. In our simulations, we removed a fixed number 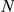 of faces at every step.

#### Force based destruction

In this method, we imposed a mechanism for destruction in which the walls carrying the highest force were broken. This is based on experimental findings that fibers in an enzymatically weakened ECM can break under the influence of mechanical forces akin to breathing [20]. As will be seen later, this mechanism sets up a specific destruction pattern in which the location of the walls to be removed next depends on the spatial history of cutting. In our simulations, we removed a fixed number 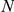 of faces at every step.

#### A mixed pattern

Here we considered a combination of the above two processes where at each step the same number of faces 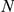 were removed at each step, but certain fraction of the faces 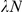, 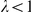 was eliminated based on force while 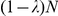 faces were eliminated randomly.

Following each step of the destruction process, the network configuration was obtained using an optimization described in the Materials and Methods section and the changes in the microscopic structure of the network were tracked by recording the volume of every cell in the network. The corresponding change in the macroscopic mechanical properties of the network was also obtained by calculating the bulk modulus, 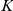, which indicates the ability of material to resist a small uniform expansion. A typical network considered had 512 cells, 1728 faces and 8856 springs.

### Structural changes

**Figure 2:**
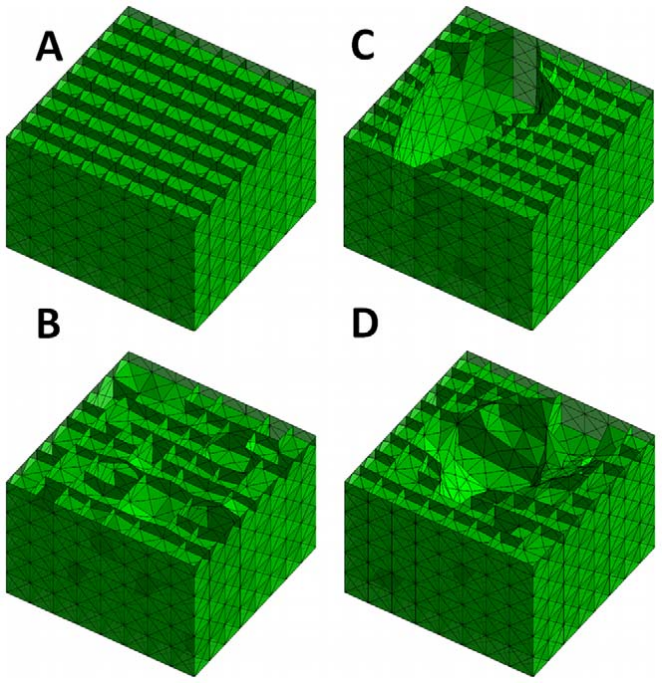
Network of cubic cells with the top removed to reveal their internal structure. (A) Initial geometry (B) Result of the random destruction pattern (C) Result of the force-based destruction pattern and (D) Result of the mixed destruction pattern. Note that networks B–D have the same drop in Bulk modulus, 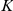, but their internal structure is very different.

Fig. 2A shows a “cut-away” view of a pre-stressed cubic network which serves as the initial network from which faces were removed during the simulation of tissue destruction in emphysema. Fig. 2B, Fig. 2C and Fig. 2D show three different structures that emerged after random cutting, force-based cutting and a mixed cutting pattern, respectively. Despite the different geometries, these networks have similar values of 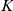.

### Decline in stiffness

**Figure 3:**
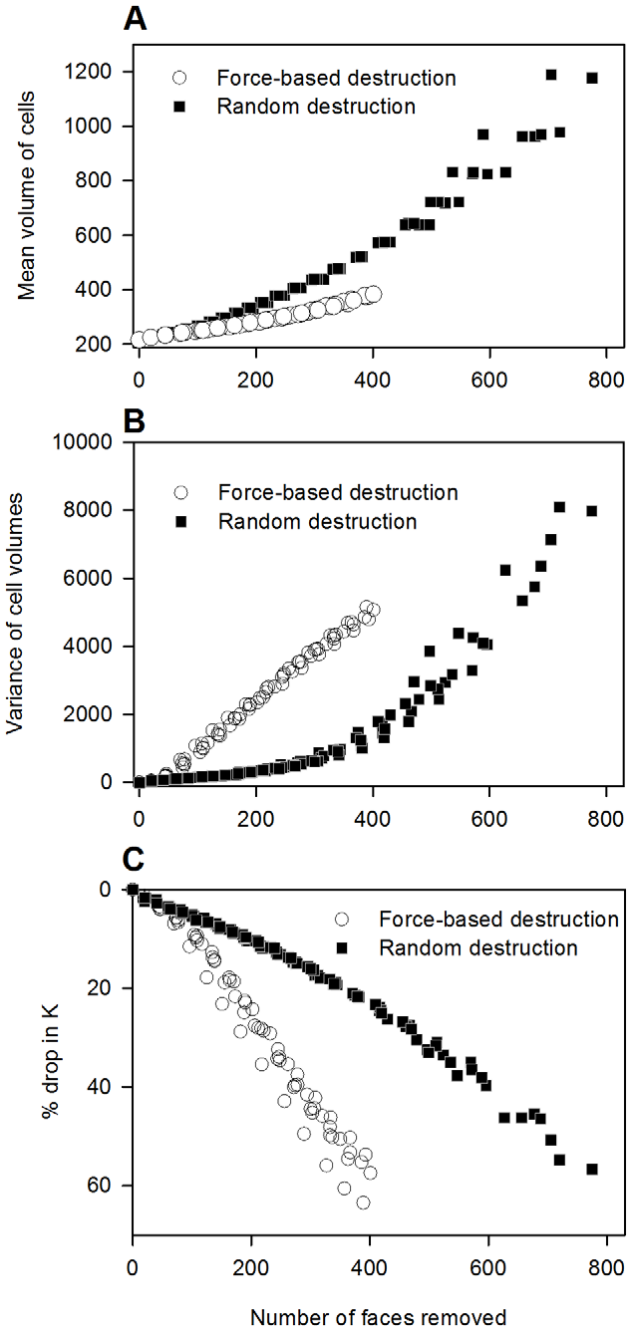
Changes in structure and function with gradual removal of faces. (A) Changes in mean volume (B) Changes in variance of cell volumes (C) Changes in bulk modulus 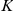.

The results in panels A and B of Fig. 3 show how the mean and variance of alveolar airspace volumes change as the number of faces eliminated from the network shown in Fig. 2A is gradually increased. For the random destruction of faces, the mean volume increases at a faster rate than for the force-based pattern; however, this trend is reversed for the variance of cell volumes with the force-based destruction resulting in a much faster increase in the variability of airspace sizes. The early rise in the variability in the force-based simulations is similar to the experimentally observed early increase in airspace size variability in animal models of emphysema [18], [21]. Fig. 3C shows the corresponding declines in 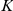: the force-based cutting method results in a fast and apparently linear decrease in 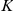 whereas in the random cutting method, the decay in 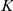 is curved and significantly slower.

### Relating changes in geometry to loss of stiffness

**Figure 4:**
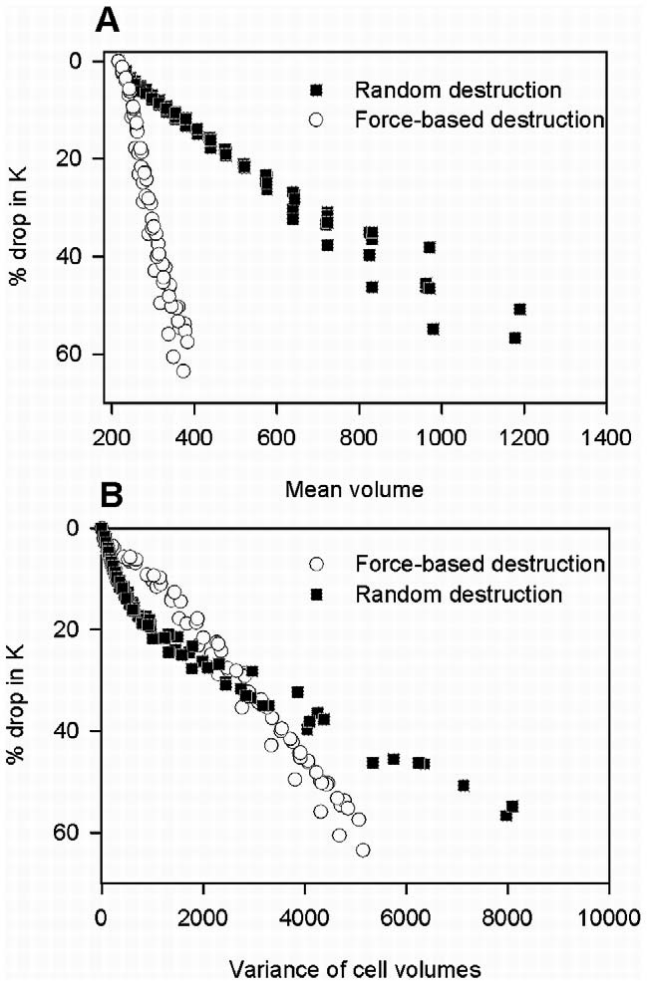
Relationship between the mean and the variance of cell volumes to the corresponding drop in bulk modulus 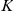 for random and force-based destruction. (A) Changes in 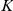 with change in mean of cell volume. (B) Changes in 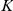 with change in variance of cell volumes.

Fig. 4A and Fig. 4B show the change in 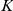 with respect to the mean and variance of cell volumes. In Fig. 4A, there is a considerable spread between the values of 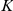 for the random and the force-based cutting implying a significantly lower macroscopic stiffness in the force-based case at the same mean cell volume. Therefore, an important result is that a given macroscopic stiffness does not correspond to a well defined mean cell volume. However, the spread decreases considerably when 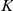 is plotted against the variance of cell volumes (Fig. 4B). This suggests that changes in higher order moments of the cell volumes may provide a better predictive relation between structural changes and decline in 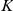.

**Figure 5:**
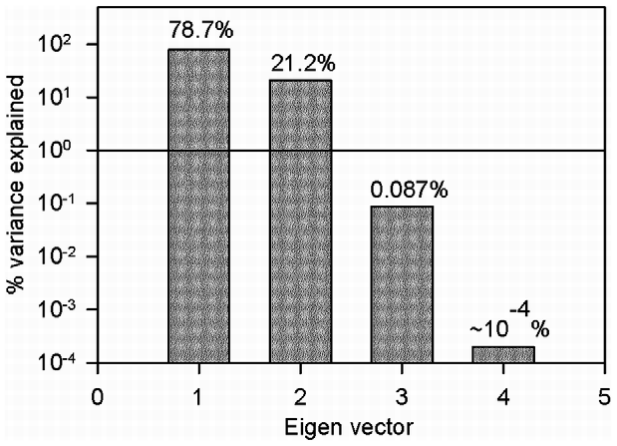
Percent variability in the data explained by each eigen vector. Note that over 99% of the variability is explained by the first two eigen vectors.

We considered four moments of the cell volume distribution: the mean 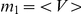 and three moments defined by 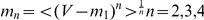. Due to the interconnected nature of the lung parenchyma, we reasoned that the way 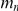 change with respect to one another during the cutting process, specifically the cross correlations between the time series 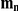, obtained from the model, would be indicative of the spatial pattern in the destruction process. To analyze the cross correlations in 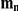, we form the matrix 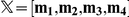, where each column in 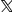 corresponds to a moment 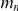 and each row corresponds to a step in the cutting process. To reduce dimensionality of the problem, we remove redundant information in 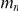 using the principal component analysis [22]–[25]. This is done by projecting 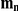 onto a new basis given by the eigen vectors of 
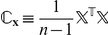
. Fig. 5 shows that 99% of the variability in 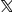 is explained by the first two eigen vectors, 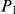 and 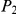 of 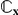, so that we only need to consider 2 dimensional data given by the projection of 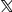 onto the subspace defined by 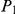 and 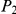. We will refer to this new transformed data as 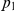 and 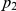. Note that 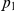 and 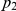 are merely the first four moments of the cell volumes projected onto a 2D subspace.

**Figure 6:**
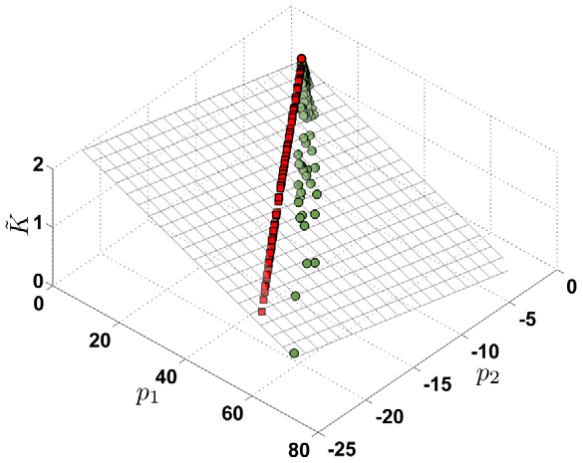
Normalized values of bulk modulus, 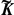 from force-based (red squares) and random destruction (green circles) plotted against the structure variables 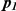 and 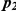. Note that the data points for both simulations lie on a single plane. The plane shown is a minimum mean square error fit to Eq. 1.

We now proceed to obtain structure-function relationships using the transformed variables 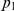 and 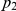. Fig. 6 shows the decline in 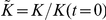 plotted against 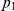 and 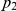 for random (green circles) and force-based (red squares) destruction patterns. Also shown in Fig. 6 is the minimum mean square error fit of the equation
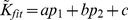
(1)to the data obtained from the random and force-based simulations. Note that a single equation was able to fit both destruction patterns. The parameters of the fit were 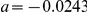, 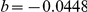, 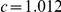.

**Figure 7:**
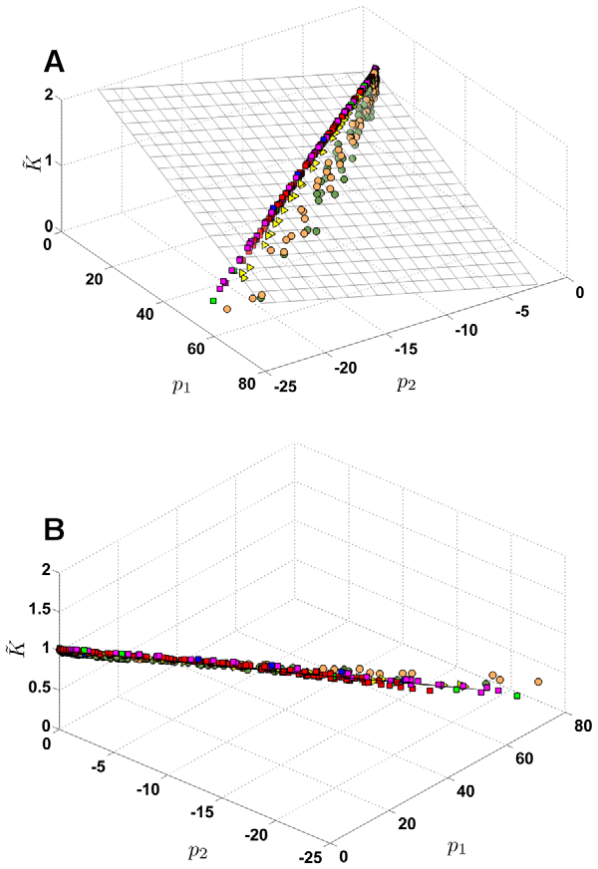
(A) The decline in 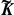 **plotted as a function of** 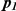 and 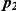 for different cutting methods. (B) shows the side view of the plane. The plane shown here is not a new fit, but is the same plane as shown in Fig. 6 which fits data from new simulations reasonably well. Data shown here correspond to force-based cutting simulations on a 14-hedral network (blue squares), a network with nonlinear springs (green squares), network at 1.5 times higher pre-stress compared to those shown in Fig. 6 (magenta squares). Simulation of mixed cutting patterns (yellow triangles) and Random cutting at higher pre-stress (orange circles). The red squares and the green circles are the same ones shown in Fig. 6 and correspond to force-based and random cutting simulations respectively.

To test our hypothesis that it is the pattern of destruction that determines changes in microscopic structure and decline in function, in Fig. 7 we plot the moments 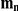 from independent simulations of force-based destruction projected on to the same 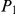, 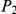 basis vectors as shown in Fig. 6. First, we considered force-based destruction on a network where the prestrain imposed was 1.5 times higher than the original set (magenta squares), we then considered a force-based destruction on a network made up of 14-hedral unit cells (blue squares) and finally we considered a nonlinear spring network where the springs developed force 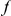 in response to strain 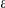 as 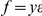 + 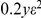, 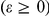. Although we only tested one particular nonlinear force-strain relation, the destruction pattern does not depend on 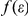 since the order in which springs are chosen for removal in the force-based cutting should not change for monotonously increasing 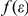. The trajectory of (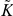, 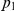, 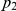) in all these cases lie very close to the original force-based cutting simulations (red squares). We have also added to the plane, data from a random cutting simulation with the initial network strained to a value 1.5 times higher than the original set (orange circles). Finally, we also considered a simulation with a mixed cutting pattern with 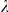  = 0.1 (yellow triangles). In all these cases, the trajectories of (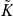, 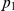, 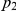) are well approximated by the plane defined by Eq. 1.

**Figure 8:**
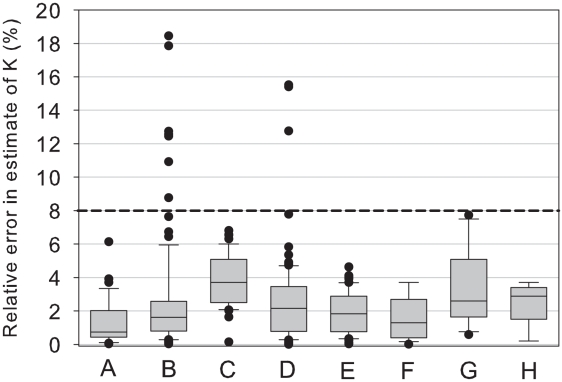
Box plot of the relative error in estimating 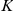 from structure. The error is expressed as percentage of the real value of 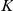. The simulations corresponds to (A) Mixed cutting pattern, (B) Random Cutting at a higher pre-strain, (C) Force-based cutting at a higher pre-strain, (D) Random cutting at a lower pre-strain, (E) Force based cutting at a lower pre-strain, (F) Force-based cutting on smaller size system with 128 cells, (G) Force-based cutting on system with 14-hedral cells, (H) Force-based cutting on system with nonlinear stress-strain relationship. Note that in all the correlated cutting mechanisms (mixed and force-based), the maximum error is less than 8%. In all the simulations, the median error was less than 4%.

To examine the how well decline in stiffness can be estimated from structural measurements, we calculate the relative estimation error given by
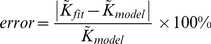
(2)where 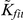, given by Eq. 1, is an estimate of stiffness from structural measurements and 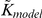 is the actual measured value. In order to identify the destruction patterns in which structural changes do not yield information about decline in stiffness (i.e., simulations with maximum error), we examine the box plot of estimation error for each destruction pattern (Fig. 8). We found that when the destruction pattern is independent (spatially random), the estimation of decline in stiffness from structure lead to very high prediction errors (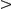
15%). However, when the pattern of destruction was spatially correlated (mixed and force based), the maximum error was less than 8% and the median error was less than 4%.

## Discussion

Traditionally, emphysema is subdivided into two major categories based on the location of destruction within the pulmonary acinus [26]. In centrilobular emphysema, which is more common and often associated with smoking, the destruction occurs mainly in the distal part of the proximal acinus. In panacinar emphysema, which is associated with 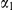 antitrypsin deficiency, more of the destruction occurs in the distal regions. At the scale of the whole lung, centrilobular emphysema shows signs of tissue destruction in the upper zones of the lung (the upper lobe and the superior segment of lower lobe) while in panacinar emphysema the destruction mostly involves lower zones and the anterior margins of the lung. At the microscopic scale, these two categories have different appearance on histological sections [14]. Further, they have also been shown to have different functional properties [27]. Saetta et al. [15] found that when human emphysema patients were classified into four categories based on patterns observed in 2D histological images ranging from a very homogeneous destruction pattern to a highly heterogeneous pattern, the subjects showed a significant difference in static compliance. These findings suggest that there is a strong link between the patterns of destruction at the microscopic scale, the geometry observed on histological sections and macroscopic lung function. Interestingly however, Saetta and coworkers found no differences in the mean linear intercept between the different groups in their study. A similar lack of correlation between tissue structure, as quantified by the mean linear intercept, and lung compliance has also been noted in animal models of emphysema [16].

In this study, we found that the decline in lung tissue stiffness was significantly influenced not only by the amount of tissue loss, but also by the spatial pattern of the destruction process (see Fig. 3C), whereby to achieve the same 60% drop in 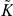, nearly twice as many faces had to be removed in the random destruction as compared to the force-based destruction. This conclusion is in agreement with previously published observations from 2D models [10]. We also found that these two cutting methods resulted in very different geometries (Fig. 2), with the correlated destruction leading to more heterogeneous structures. This finding has important implications on the characterization of emphysema from histological sections. Our results indicate that the heterogeneity in microscopic structure observed in the early stages of emphysema [18], [21], is an indicator of the pattern of destruction and hence is also indicative of the extent of decline in tissue stiffness. One possible reason for the disconnect between structure and compliance noted above maybe due to the fact that currently accepted standards for quantifying structural changes in emphysema do not account for the patterns in tissue destruction [28].

In a recent study [29], we examined the relation among alveolar structure, tissue composition and lung function. Specifically, respiratory compliance C was correlated to biochemical and structural parameters of the mouse lung before and after elastase-induced emphysema. Interestingly, C did not correlate with bulk measures of soluble type I collagen, type III collagen or elastin. There was, however, a strong association between C and the mean equivalent diameter of airspaces (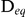), and a much stronger relation between C and the area weighted mean diameter (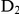) with R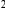 values of 0.675 (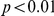) and 0.933 (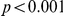), respectively. Since 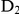 includes higher order moments of the distribution of diameters [30], it is highly sensitive to structural heterogeneities and hence patterns. Thus, there is now experimental data showing that it is not the mean airspace size, but its heterogeneity that determines function in agreement with the network analysis we presented here.

Several previous publications have used different 3D models to examine the elasticity of normal lung tissue. Kimmel and Budiansky [31] employed a dodecahedral model to calculate elastic moduli for small deformations about a state of uniform expansion. More sophisticated models have later been proposed to examine non-uniform, large deformations [32]–[34]. Denny and Schroeter [33] also examined, using 3D models, changes in tissue elasticity when the relative amount of collagen versus elastin is perturbed as happens in the early stages of emphysema. However, to the best of our knowledge, the change in tissue elasticity associated with destruction of alveolar walls and its relation to structural changes have not been examined thus far.

In order to examine how our results compare to observations in real emphysema, it is important to consider the factors that influence 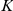 in real lung tissue and the limitations of the present model. In this study, we considered a small block of tissue far away from the major airways and devoid of ducts. The tissue network of the lung is usually classified into 3 interdependent compartments: a peripheral tissue system consisting of the pleural membrane and the interlobular membranes, an axial system which forms the alveolar ducts and surrounds the mouth of alveoli where they join the ducts and the fiber network that forms the alveolar septa [35], [36]. Since the predominant structural change in emphysema is destruction of alveolar septa [37], [38], it is only this part of the lung tissue that we considered in this study.

In our model, we only considered the recoil forces provided by the protein fibers that make up the ECM. However, the walls of the alveoli are coated with a liquid layer that provides surface tension at the air liquid interface. The value of surface tension is lowered by surfactant released by epithelial cells [39]. Surface tension forces act in two ways, they provide a recoil pressure [40] and, additionally, they distort the parenchymal geometry thereby providing an indirect contribution to the recoil forces [41]. The problem of how surface tension changes in emphysema may affect functional properties has been studied using models [42]. However, a recent experimental study suggested no change in surface tension in the lung due to emphysema [43]. Since the airspace sizes are generally larger in emphysema, surface forces likely decrease and the effect of surface tension may not be important in affecting the process of tissue destruction. Hence, we neglected the contribution of surface tension to elastic recoil.

We simulated tissue destruction in a pre-strained network with the outer boundary fixed. In this case, during the destruction process, the total volume of the network is conserved. If we changed the boundary condition to a pressure boundary condition, which is perhaps more realistic, then, as tissue is destroyed, the whole network would expand outward thereby increasing the total volume. In this case, a network with such a boundary condition would distribute stresses differently after an alveolar wall is destroyed and the pattern of force-based destruction would be somewhat different from a fixed boundary condition. Nevertheless, the pressure boundary is also not fully consistent with physiology since the distending stress around the lung is maintained by the balance of the nonlinear chest wall elasticity and the changing lung recoil during the progression of emphysema. These issues represent significant additional computational challenges and will have to be examined in future studies.

Before concluding, we note the following implication of the modeling results to disease progression. As Fig. 3C demonstrates, the pattern of tissue destruction has a significant impact on the rate of stiffness decline. Whether or not correlated patterns develop depends on the presence or absence of mechanical forces. In the normal lung, mechanical forces are present everywhere due to the negative pleural pressure surrounding the lung. However, these forces are not sufficient to rupture the tissue. In the diseased lungs, enzymatic damage weakens the tissue and lowers its failure threshold allowing mechanical forces of breathing to rupture the alveolar septal walls [20]. Thus, a lack of heterogeneity in the emphysematous tissue structure implies that mechanical forces are small or not dominating the destruction process and, consequently, the progressive nature of the disease must be driven by biological mechanisms that produce strong enzymatic tissue digestion. This may have further implication for treatment since enzymatic activity may be attenuated pharmacologically whereas eliminating mechanical forces is not feasible since it would lead to lung collapse.

To summarize, we have developed a 3D computational model and a general framework to relate structural changes characterized by the cell volume distribution to functional changes. Our simulations demonstrate that different destruction mechanisms lead to grossly different microscopic destruction patterns which in turn result in different cell volume distributions and macroscopic declines in function for the same amount of tissue loss. It is therefore important to consider both the total amount as well as the spatial history of the destruction process in an attempt to relate structure to function. Further, our results suggest that changes in variability and higher order moments of the alveolar dimensions are not only important in determining changes in function but observing the corresponding structural patterns may also provide insight into the mechanism of disease progression. Finally, appropriate application of the uncovered structure-function relations to real lungs may in the future help evaluate the efficacy of therapies or novel drugs.

## Materials and Methods

We consider a cuboidal block of lung tissue which we model as a 3D structure formed by tiling space filling polyhedra, each of which represents an idealized alveolus. In this study, we used two kinds of space filling polyhedra: the cube, the simplest platonic space filling solid and the 14-hedron, a solid with 8 regular hexagons and 6 square faces [32], [34], [44], [45]. The initial geometry of a block of lung tissue composed of either cubes or 14-hedra is shown in Fig. 1.

### Elastic properties

To model the elastic properties of the alveolar wall, each face of an idealized alveolus has linear hookean springs connecting its vertices to its centroid (Fig. 1A and Fig. 1C). Additionally, springs are also placed along the edges of the polyhedron. These face and edge springs represent the combined effect of collagen and elastin fibers that are considered to be the two major force-bearing components that make up the alveolar wall. For small strains, we will assume that these springs develop a force 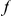 in response to an applied strain 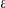 as
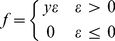
(3)


The assumption here is that all the springs are made of the same material so that the constants 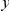 is a property of the material analogous to the Young's modulus. It should be noted that springs in the model do not support compression. Consequently, the networks shown in Fig. 1 are inherently unstable and will collapse when subjected to a shear deformation. In order to stabilize the model, it is necessary to apply a pre-strain to the structure.

### Pre-strain and boundary conditions

The model is capable of being pre-strained in 3 different ways.

1. Fixed Boundary: The entire network is subjected to a uniform expansion and the vertices along the faces that make up the exterior boundary (boundary nodes) are fixed. When the entire network is stretched out uniformly, individual springs become stretched and the equilibrium configuration is then determined by minimizing the total energy of the network given by
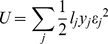
(4)where 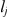
s are the unstretched lengths of the springs and the summation is carried out over all the springs in the network.

2. Force boundary: To each boundary node, 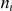, an external force 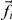 is applied. The set of forces 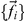 determine the boundary condition. The equilibrium configuration is calculated by minimizing the free energy, 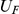 which takes into account the internal energy of the spring network 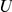 and the work done by the external forces 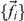. The minimization is carried out on 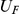, changes in which are defined by:
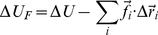
(5)where 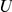 is given by Eq. 4 and 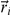 is the position vector of the boundary node 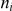.

3. Pressure Boundary: A negative external pressure (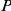) can be applied to the entire network and the equilibrium configuration can be calculated by minimizing the free energy 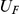 which accounts for the total internal energy of the network and the work done by the applied pressure 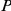. The change in free energy 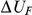 is given by
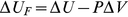
(6)where 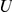 is given by Eq. 4 and 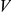 is the current volume of the expanding network. In the model, a face is composed of a set of non-overlapping triangles, so that the pressure acting on a triangular facet with outward normal area vector 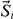 generates a force 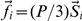 to act on the three nodes that make up the triangular facet. The network is then allowed to reach an energy minimum as described below. As the network equilibrates, the change in geometry of the network causes the vectors 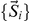 to change. The new set of area vectors 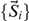 and forces on the boundary nodes 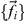 are updated. The minimization procedure is repeated until the change in 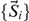 between two successive minimization steps falls below a preset error bound.

#### Numerical optimization

Idered in this study had more than 5000 nodes. For such a high dimensional system, it is difficult to ascertain the exact nature of the energy surface. Starting with the assumption that Eq. 4–6 lead to an energy surface with multiple local minima, the simulated annealing algorithm [46], [47] was first used to minimize the energy of the network. This technique uses a control parameter usually referred to as temperature (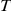) which is set to a high value initially. The system is perturbed by moving every node by a small amount and the resulting configuration is accepted based on the probability 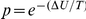 where 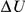 is the associated change in free energy. These steps are repeated until the system reaches thermal equilibrium at which point the temperature is reduced. In our simulations, we found that the global minimum achieved using this method was the same regardless of the starting temperature. Since zero temperature minimization corresponds to moving every node in the opposite direction of the local gradient vector of the energy surface, it was concluded that the energy surface is convex with only one minimum and the energy minimization was done using a gradient-descent algorithm. The equilibrium criteria were set based on the condition that both the magnitude of the maximum resultant force in the network and the magnitude of the mean resultant force in the network are below certain specified thresholds. While the former is a stricter condition for local deformations, the latter is the stricter condition for uniform deformations.

Once faces are removed from the network, the equilibrium configuration can only be calculated numerically by minimizing the total free energy of the system. However, in the case of an intact network, it is possible to derive analytic expressions for the elastic moduli and compare them to the values obtained from the computational model. To verify that our computational model, in the supplementary material (see supplementary information file Text S1), we considered two simple cases (1) A single cube for which the equilibrium configuration after applying a pressure change or a shear deformation can be easily calculated and (2) An intact cubic network of consisting of several cubes where we compare theoretical values of 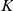 to those obtained from the numerical model.

### Measurements

Changes in the microscopic structure of the network as a result of tissue destruction are tracked by recording the volume of every cell in the network. The change in macroscopic mechanical properties of the network are also tracked by measuring the bulk modulus, 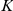 which indicates the ability of the material to resist a small uniform expansion and the shear modulus, 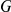 which is a measure of the materials ability to resist small iso-volume shape distortions.

#### Calculating cell volumes

Initially, all the cells in the network consist of convex polyhedra. However with the destruction of faces, cells can assume non-convex configurations. To calculate the volume of non-convex polyhedra, we use the Gauss divergence theorem which states that the volume integral of the divergence of a vector field 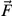 over a closed region 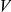 is equal to the surface integral over its bounding surface 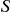.
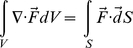
(7)where 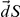 is the outward normal vector to the surface 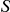.

By setting the vector field 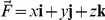, Eq. 7 becomes
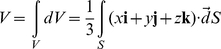
(8)


For a non-convex polyhedral cell enclosed by non overlapping triangular facets, Eq. 8 states that the volume of the cell is simply the sum of signed volume of tetrahedra formed by a triangular facet with area vector 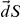 as its base and the origin as its apex.

#### Calculating the bulk modulus

The bulk modulus, 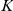 can be measured from the model under any boundary condition and does not require the bounding box to be cuboidal. To measure 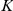, we apply a small pressure change 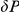 to the network. If the network is under a fixed boundary condition, before applying 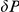, we switch to a force boundary condition by adding an external force to each boundary node which has the same magnitude as the resultant force acting on that node but acts in the opposite direction. The boundary nodes are then allowed to move freely. After equilibrating the network, the resulting volume expansion 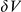 is measured and 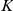 can be calculated as 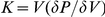.

### Principal Component Analysis

Principal Component Analysis (PCA) also known as the Karhunen-Loève transform or the Hotelling transform [22]–[25] is a common method for removing correlations in an input data set. This is done by projecting the input data onto a new basis which is derived from the original data set. Let 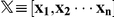 represent the original data set with the vector 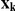 representing different observations of 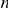 correlated input variables. We first form the covariance matrix 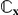 given by
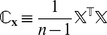
(9)

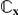
 is a square matrix whose diagonal elements 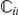 are the variance of the i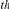 variable in 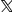 and 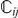 are the covariance of variables corresponding to 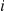 and 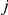. Next we find the eigen vectors 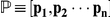 and eigen values 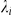 of 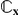. The original data set, 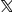, can be transformed to a new data set by projecting 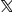 on to the new basis defined by the eigen vectors 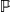. The advantage in doing this is that, depending on the level of correlation in 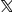, 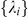 usually decrease extremely fast and only the first few vectors in the new basis of 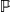 need to be considered, so that the new projected data is usually of lower dimensionality than the original set.

## Supporting Information

Text S1 In this supplement, we verify the accuracy of our computational model by comparing analytically calculated values of elastic moduli of a single cube as well as the bulk modulus of an intact cubic network with values obtained from the computational model.
(0.23 MB PDF)
