# Supplementary material for: Linking Microscopic Spatial Patterns of Tissue Destruction in Emphysema to Macroscopic Decline in Stiffness Using a 3D Computational Model
Source: PLoS Comput Biol. 2011 Apr 21;7(4):e1001125. doi: 10.1371/journal.pcbi.1001125 (PMC3080851; doi:10.1371/journal.pcbi.1001125)
Supplement: Text S1 — In this supplement, we verify the accuracy of our computational model by comparing analytically calculated values of elastic moduli of a single cube as well as the bulk modulus of an intact cubic network with values obtained from the computational model. (0.23 MB PDF) [file pcbi.1001125.s001.pdf]

Text S1: Supplementary Information File

Verifying the computational model

Once faces are removed from the network, the equilibrium configuration can only be calculated numerically by minimizing the total free energy of the system. However, in the case of an intact network, it is possible to derive analytic expressions for the elastic moduli and compare them to the values obtained from the computational model. Specifically, we considered two simple cases (1) A single cube for which the equilibrium configuration after applying a pressure change or a shear deformation can be easily calculated and (2) An intact cubic network of consisting of several cubes where we compare theoretical values of K to those obtained from the numerical model.

Elastic properties of a single cell

In this section, we calculate the elastic moduli of a single cell and compare the results with those obtained from the numerical model.

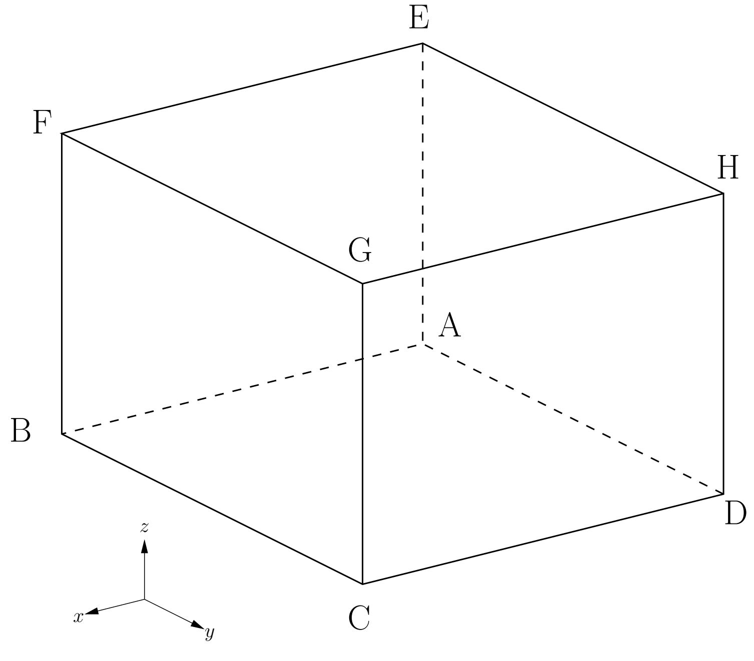

Figure 1. Single cube model

We consider a cube with its center at $\{0,0,0\}$ and corners at $\{\pm a/2, \pm a/2, \pm a/2\}$. Let P, Q, R, S, T and U denote the face centers of the faces ABCD, ABFE, BCGF, CDHG, DAEH and EFGH, respectively (see Fig. 1 in text S1). The corner A is connected to the corners B, D and E by edge springs and to face centers P, Q and T by face springs. The length ℓ of the edge springs and face springs are given by:

$$\ell_{AB} = \ell_{AD} = \ell_{AE} = a \tag{1a}$$

$$\ell_{AP} = \ell_{AQ} = \ell_{AT} = a/\sqrt{2} \tag{1b}$$

For the present purpose we will assume that every spring has a force-strain relationship of the form specified in Eq. 3 with $y = Y$. We apply a deformation gradient tensor $\mathbb{U}$ to this cube such that the position vector corresponding to each node $\vec{r}$ transforms to $\vec{r}^{\,\prime}$ as :

$$\vec{r}^{\,\prime} = \mathbb{U} \cdot \vec{r} \tag{2}$$

Under the deformation $\mathbb{U}$, the resultant force on A can be written as,

$$\begin{aligned} \vec{f}_A &= \sum_{i\in\{\mathrm{B,D,E}\}} \frac{Y}{a} \left(|\vec{r}_i^{\,\prime} - \vec{r}_A^{\,\prime}| - a \right) \frac{\vec{r}_i^{\,\prime} - \vec{r}_A^{\,\prime}}{|\vec{r}_i^{\,\prime} - \vec{r}_A^{\,\prime}|} \\ &+ \sum_{i\in\{\mathrm{P,Q,T}\}} \frac{\sqrt{2}Y}{a} \left(|\vec{r}_i^{\,\prime} - \vec{r}_A^{\,\prime}| - \frac{a}{\sqrt{2}} \right) \frac{\vec{r}_i^{\,\prime} - \vec{r}_A^{\,\prime}}{|\vec{r}_i^{\,\prime} - \vec{r}_A^{\,\prime}|} \end{aligned} \tag{3}$$

and the resultant force on the face center P is given by

$$\vec{f}_P = \sum_{i\in\{\mathrm{A,B,C,D}\}} \frac{\sqrt{2}Y}{a} \left(|\vec{r}_i^{\,\prime} - \vec{r}_P^{\,\prime}| - \frac{a}{\sqrt{2}} \right) \frac{\vec{r}_i^{\,\prime} - \vec{r}_P^{\,\prime}}{|\vec{r}_i^{\,\prime} - \vec{r}_P^{\,\prime}|} \tag{4}$$

Note that Eqs. 3 and 4 are valid for any deformation tensor $\mathbb{U}$.

Pre-Strain: We prescribe an isotropic pre-strain by setting $\mathbb{U} = \mathbb{U}_{\mathrm{ps}}$ given by

$$\mathbb{U}_{\rm ps} \equiv \begin{pmatrix} 1+\epsilon & 0 & 0 \\ 0 & 1+\epsilon & 0 \\ 0 & 0 & 1+\epsilon \end{pmatrix} \tag{5}$$

For $\mathbb{U} = \mathbb{U}_{\rm ps}$,

$$\vec{r}_i^{\,\prime} - \vec{r}_j^{\,\prime} = (1+\epsilon)\,(\vec{r}_i - \vec{r}_j) \tag{6}$$

and thus

$$\vec{f}_A = \sum_{i\in\{\mathrm{B,D,E}\}} \frac{Y\epsilon}{a}\,(\vec{r}_i - \vec{r}_A) + \sum_{i\in\{\mathrm{P,Q,T}\}} \frac{\sqrt{2}Y\epsilon}{a}\,(\vec{r}_i - \vec{r}_A) \tag{7}$$

$$= \frac{Y\epsilon}{a}\left(\frac{1+\sqrt{2}}{a}\right) a\,\{1,1,1\} \tag{8}$$

and

$$\vec{f}_p = \sum_{i\in\{\mathrm{A,B,C,D}\}} \frac{\sqrt{2}Y\epsilon}{a}\,(\vec{r}_i - \vec{r}_P) \tag{9}$$

$$= 0 \tag{10}$$

On the face ABCD, the outward normal is given by $\hat{n}_{\rm ABCD} = \{0,0,-1\}$. The total external force normal to this face is

$$f_{\rm ABCD} = \sum_{i\in\{\mathrm{A,B,C,D,P}\}} \left(-\vec{f}_i \cdot \hat{n}_{\rm ABCD}\right) \tag{11}$$

$$= 4\,Y\epsilon\left(1+\sqrt{2}\right) \tag{12}$$

The area of the face ABCD is

$$A_{\rm ABCD} = (1+\epsilon)^2\,a^2 \tag{13}$$

and thus the effective pressure on the face is

$$P = f_{\text{ABCD}}/A_{\text{ABCD}} = 4\,\frac{Y\epsilon}{a^2}\,\frac{1+\sqrt{2}}{(1+\epsilon)^2} \tag{14}$$

and the volume of the cube is

$$V = a^3\,(1+\epsilon)^3 \tag{15}$$

Bulk modulus K: From Eqns. 14 and 15, the bulk modulus, K can be calculated as the derivative $\frac{\partial P}{\partial V/V}$:

$$K = V\frac{\partial P}{\partial V} = V\frac{\partial P/\partial \epsilon}{\partial V/\partial \epsilon} = 4Y\,\frac{\left(1+\sqrt{2}\right)}{3a^2}\,\frac{(1-\epsilon)}{(1+\epsilon)^2} \tag{16}$$

The change in K with ϵ calculated from Eq. 16 is plotted as lines in Fig. 2 of text S1, with the data from the computational model superimposed on it. It can be seen that the computational model and the theoretical calculation are in agreement.

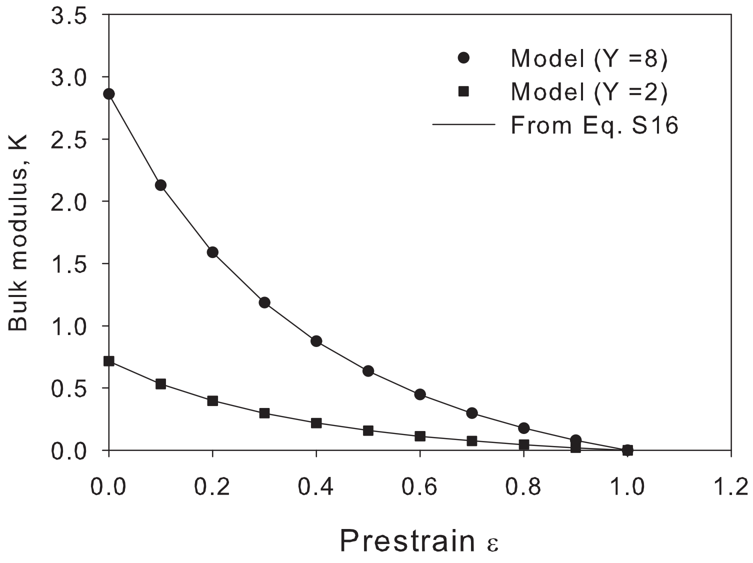

Figure 2. Bulk modulus K of a single cube as a function of pre-strain ϵ.

Shear modulus G: The G can be calculated for the pre-stressed network applying a deformation specified by

$$\mathbb{U}_{\text{sm}} \equiv \begin{pmatrix} 1 & 0 & \gamma \\ 0 & 1 & 0 \\ 0 & 0 & 1 \end{pmatrix} \mathbb{U}_{\text{ps}} \tag{17}$$

Note that $\mathbb{U}_{\text{sm}}$ is applied on the unstretched network. From Eqs. 3 and 4, the shear modulus, G can be calculated as the change in shear stress τ for an applied shear strain γ in the limit when $\gamma \to 0$:

$$G = \left.\frac{\partial \tau}{\partial \gamma}\right|_{\gamma \to 0} = \begin{cases} \frac{Y}{a^2} \frac{\left(\sqrt{2}+4\left(1+\sqrt{2}\right)\epsilon\right)}{(1+\epsilon)^2} & \text{if } \epsilon > 0 \\ \\ \frac{Y}{\sqrt{2}a^2} & \epsilon = 0 \end{cases} \tag{18}$$

It is interesting to note that G is discontinuous and nonzero at zero prestress. The change in G with ϵ calculated from Eq. 18 is plotted as solid lines in Fig. 3 of text S1 with the data from the computational model superimposed on it. It can be seen that the computational model and the theoretical calculation are in perfect agreement.

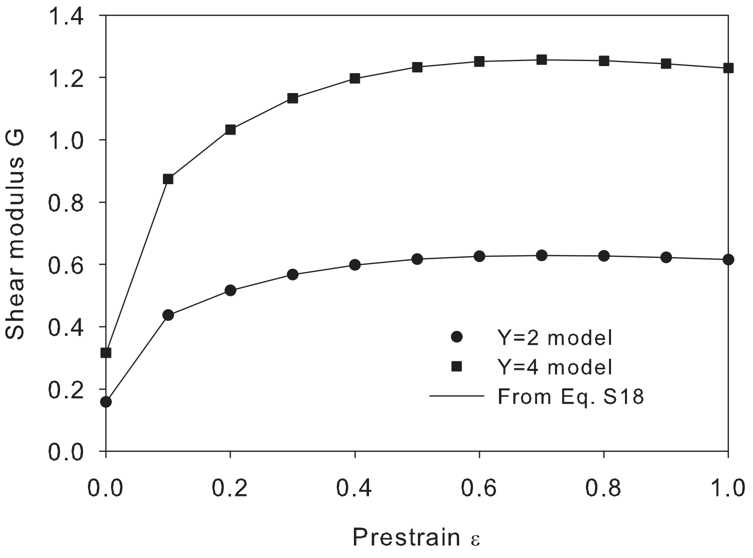

Figure 3. Shear modulus G of a single cube as a function of pre-strain ϵ.

Young's modulus E: can be calculated from the pre-stressed network applying a deformation specified by

$$\mathbb{U}_{\rm ym} \equiv \begin{pmatrix} 1+\xi & 0 & 0 \\ 0 & 1 & 0 \\ 0 & 0 & 1 \end{pmatrix} \mathbb{U}_{\rm ps} \tag{19}$$

Note that $\mathbb{U}_{\rm ym}$ is applied to the unstretched network. From Eqs. 3 and 4, E can be calculated as:

$$E = \left.\frac{\partial \tau}{\partial \xi}\right|_{\xi \to 0} = \frac{2Y\left(2+\sqrt{2}+2\left(1+\sqrt{2}\right)\epsilon\right)}{a^2\left(1+\epsilon\right)^2} \tag{20}$$

The change in E with ϵ calculated from Eq. 20 is plotted as solid lines in Fig. 4 of text S1 with the data from the computational model superimposed on it. It can be seen that the computational model and the theoretical calculation are in agreement.

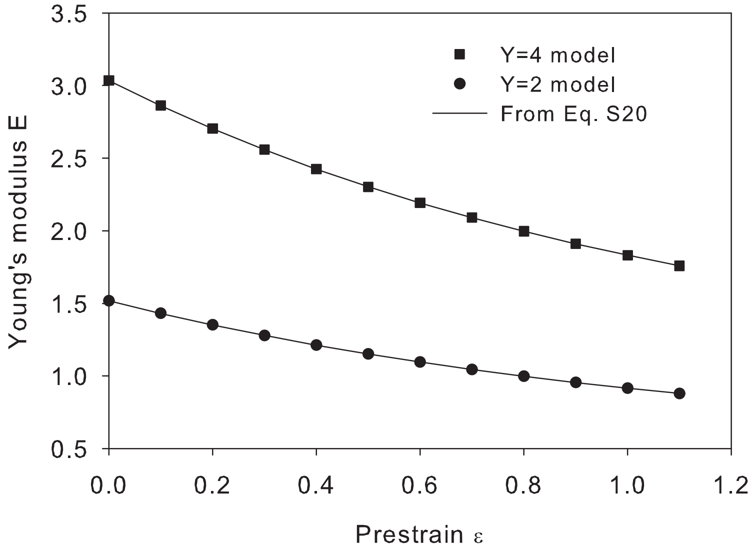

Figure 4. Young's modulus E of a single cube as a function of pre-strain ϵ.

Calculating K for a network with multiple cells

In this section, we calculate the bulk modulus K of a three dimensional cubic network with N_C cubes, N_F face springs and N_E edge springsunder a pre-strain of ϵ. We assume that all springs in the network

also experience the same strain ϵ and develop a force f in response to applied strain ϵ as $f = Y\epsilon$. The potential energy U and the volume V of the cubic network are given by:

$$U = \frac{1}{2}aN_E Y\epsilon^2 + \frac{1}{2\sqrt{2}}aN_F Y\epsilon^2$$
$$V = a^3 N_C(1+\epsilon)^2 \tag{21}$$

where a is the unstretched length of a side of each cubic cell in the network. Since the network is perfectly elastic, the potential energy of the network must be matched by the work done by an external positive pressure P. Therefore,

$$P = \frac{\partial U}{\partial V} = \frac{(\partial U/\partial \epsilon)}{(\partial V/\partial \epsilon)} = \frac{\left(2N_E + \sqrt{2}N_F\right) Y\epsilon}{6a^2 N_C\,(1+\epsilon)^2} \tag{22}$$

From Eqn. 21 and Eqn. 22, we can find the Bulk modulus K as:

$$K = V\frac{\partial P}{\partial V} = V\frac{(\partial P/\partial \epsilon)}{(\partial V/\partial \epsilon)} = \frac{\left(2N_E + \sqrt{2}N_F\right) Y\,(\epsilon - 1)}{18a^2 N_C\,(1+\epsilon)^2} \tag{23}$$

In Fig. 5 of text S1 , we show K from the computational model plotted along with K calculated using Eq. 23 for a network with 343 cubic cells for different values of ϵ. Note that there is perfect agreement between the calculation and the numerical results from the model.

Using Eq. 23, we can also find the Bulk modulus of an unstretched network for different N_C, N_F, N_E values as:

$$K = \frac{\left(2N_E + \sqrt{2}N_F\right) Y}{18a^2 N_C} \tag{24}$$

In figure 6 of text S1 , we consider a cubic bounding box with a fixed volume and calculate the change in K as the number of cells used to tesselate the fixed volume is increased. The results from the computational model are plotted along with K calculated using Eq. 24 .

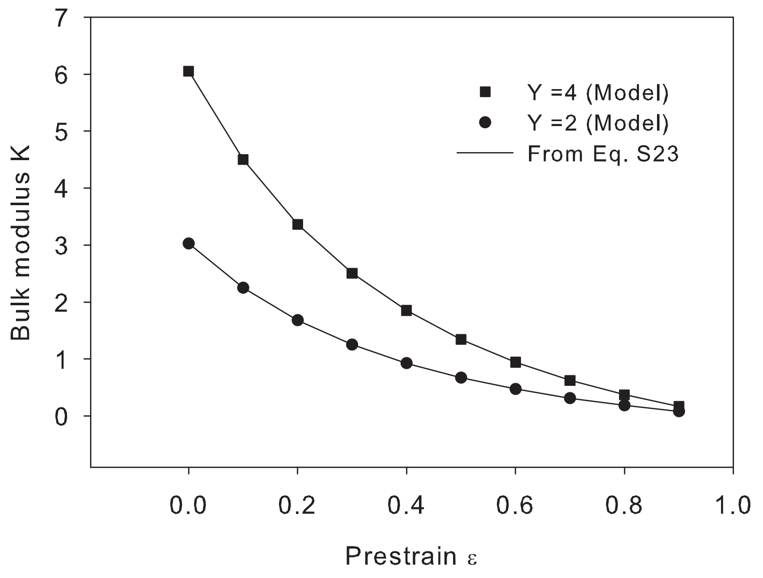

Figure 5. **Change in K with pre-strain for an intact cubic network with 343 cells.**

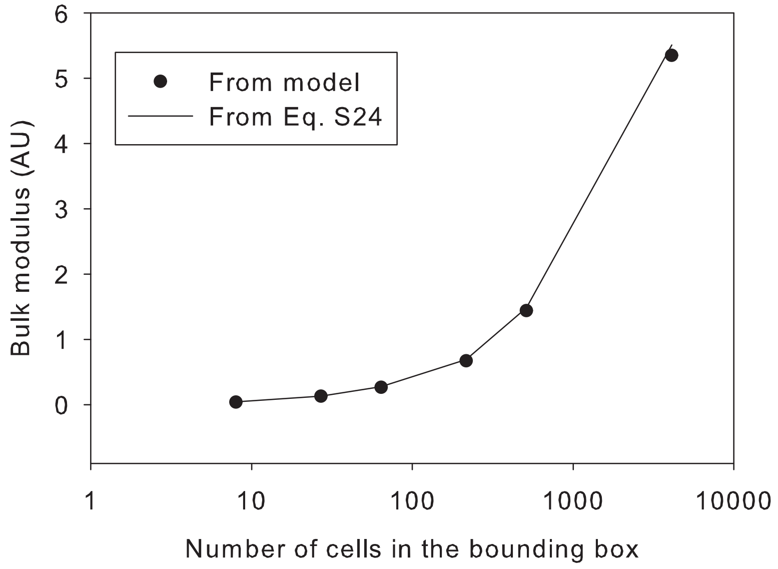

Figure 6. **K for an unstretched cubic networks with different cell densities.**
